# CD98hc has a pivotal role in maintaining the immuno-barrier integrity of basal layer cells in esophageal epithelium

**DOI:** 10.1186/s12935-021-02399-5

**Published:** 2022-02-22

**Authors:** Hao Ye, Xiang Li, Jing Lin, Peng Yang, Min Su

**Affiliations:** 1 Institute of Clinical Pathology, Guangodng Provincial Key Laboratory of Infectious Disease and Molecular Immunopathology, Shantou University Medical College, No. 22 Xinling Road, Shantou, 515041 Guangdong People’s Republic of China; 2 The Judicial Critical Center, Shantou University Medical College, No. 22 Xinling Road, Shantou, 515041 Guangdong People’s Republic of China

**Keywords:** Esophageal squamous cell carcinoma, Carcinogenesis, Chronic inflammation

## Abstract

**Objectives:**

The current study aims to find the linker between esophageal epithelial carcinogenesis and chronic inflammation and the origin of hyperproliferative cells in precancerous lesions of esophageal squamous cell carcinoma (ESCC).

**Materials and methods:**

Twenty one normal esophageal tissues from cadavers and 180 paired tissues from 60 surgical resected ESCC specimens were utilized for immunohistochemistry staining against CK14, CK6, CD98hc and Ki67. NE6 cell line was treated with H_2_O_2_ to mimic chronic inflammation microenvironment and TPA for malignant orientated transformation. Cell proliferation and CD98hc mRNA were assessed by CCK8 assay and RT-qPCR.

**Results:**

CD98hc expression was correlated with chronic inflammation severity, precancerous lesion stage, and epithelial cell proliferative activity. CD98hc expression and proliferation rate of NE6 were up regulated by low dose H_2_O_2_ treatment and long term TPA treatment. The proliferating cells in hyperplastic and dysplastic tissues could be divided into two patterns by the expression of CK14, CD98hc, CK6 and Ki67: CK14^+^CD98hc^+^CK6^−^Ki67^−^ in basal cells with CK14^−^CD98hc^−^CK6^+^Ki67^+^ in proliferating cells and CK14^+^CD98hc^+^CK6^+^Ki67^+^ in both basal cells and proliferating cells.

**Conclusions:**

Our study revealed that CD98hc was a marker of cells originated from basal cell in esophagus, ectopic expression of CD98hc in hyperplastic/dysplastic cells by chronic inflammation stimulation crippled the linkage between basal cell and basement membrane, sabotaged the integrity of the barrier in between lamina propria and epithelium, subsequentially initiate carcinogenesis.

**Supplementary Information:**

The online version contains supplementary material available at 10.1186/s12935-021-02399-5.

## Introduction

Defined as the seventh-ranked malignancies around the world, esophageal carcinoma is rated as the sixth in cancer-related deaths [1]. Esophageal squamous cell carcinoma (ESCC) is the most common histologic subtypes of esophageal carcinoma [2].

The accompany of chronic inflammation in tumorigenesis was put forward by Rudolf Virchow about 100 years ago [3]. Ulcerative colitis, a common form of inflammatory bowel illness, harbors the relevance with an added risk of colorectal cancer. The development of colorectal cancer in ulcerative colitis patients might represent featured examples of the association between inflammation and carcinogenesis [4]. CD98hc, the CD98 heavy chain encoded by SLC3A2, is a Type II transmembrane protein and covalently connected to CD98 light chains, as a functional heterodimeric large neutral amino acid transport systems [5, 6]. CD98hc is also associated with integrin β1, a major component of focal adhesion that link basal layer cell with basement membrane(BM) in epithelial tissues, thus, revising integrin signaling in controlling cell proliferation, survival, migration and epithelial cell adhesion/polarity [7, 8]. Merlin et al. harbored the conception that intestinal epithelial cell-specific CD98hc overexpression favoring intestinal inflammation; in consequence, it helps promote colitis-associated tumorigenesis in mice [9]. In previous researches, chronic inflammation was found to induce DNA damage, stimulate cellular proliferation and correlate with the severity of precancerous lesion in esophageal epithelium [10, 11]. The role of CD98hc in the crosstalk between chronic inflammation and esophageal carcinogenesis is yet to be explored.

Researches have revealed that malignant transformation of human esophageal mucosa can be modeled into a multistage process [12, 13]. Basal cell hyperplasia, dysplasia and carcinoma in situ, which were considered as precancerous lesions of ESCC [14]. For normal epithelium, Seery et al. proposed a model that previously defined esophageal basal zone (bottom 1–3 layers) could be finely divided into basal cells (cells proximate the BM) and epibasal cells (1–2 layers above basal cells) [15]. During basal cell hyperplasia, hyperproliferating basal cells could form over three layers, while in dysplasia, nonpolar cell with cellular and nuclear atypia could occupy up to the entire epithelium [16]. But studies about whether the proliferated cells were originated from basal cells or epibasal cells, remain insufficient.

In the current research, we mainly studied the expression of CD98hc in human esophageal epithelium under physiological and pathological condition and primarily explored the role of CD98hc in esophageal carcinogenesis. We utilizedCK14 to mark basal cells, CK6 to mark hyperproliferative keratinocytes and Ki67 to mark proliferating cells [17–19]. By IHC staining against CK6, CK14 and Ki67 in precancerous esophageal epithelium, we explored the origination of hyperplastic and dysplastic cells. We found that hyperplastic or dysplastic cells were either derived from basal layer or epibasal layer, and CD98hc was devotedly expressed in basal cell derived cells, accordantly, in normal esophageal epithelium, CD98hc expression was basal cell layer specific.

## Materials and methods

### Tissue samples

180 paired tumors, adjacent esophageal mucosa (within 2 cm from tumor) and distant normal mucosa (5 cm from tumor) from 60 surgical resected ESCC specimens without any prior radio- or chemotherapy were collected from the Cancer Hospital of Shantou University Medical College. 21 normal esophageal tissues at different ages in paraffin embedded tissue blocks were obtained from The Judicial Critical Center, Shantou University Medical College, written informed consents were received from relative of the deceased. The research was approved by the ethical board of Shantou University Medical College, ethic license number was SUMC-2015-15.

### Cell culture and H_2_O_2_, TPA treatment

Immortalized normal esophageal epithelial cell NE6 were a gift kindly given by Prof. George Tsao from Hong Kong University in 2008. NE6 has been authenticated by DNA STR analysis in June of 2020 in the lab of IGE BIOTECH INC., LTD. (Guangzhou, China), resulted as a unique and none contaminated cell line. NE6 were cultured in mixed medium with an equal amount mixture of Epilife and Defined Keratinocyte-SFM (Gibco, Thermo Fisher), supplemented with growth factor (Def Ker Growth Supp 1 ml, Gibco, Thermo Fisher) and penicillin/streptomycin. Cultured at 37 °C in humidified incubator with 5% CO_2_.

NE6 were treated by 1 μM H_2_O_2_ for 48 h, fresh culture medium containing 1 μM H_2_O_2_ was changed every 24 h. Cell proliferation was determined by CCK8 assay after 48 h. RNA and protein were extracted at the same time.

TPA (12-O-Tetradecanoylphorbol-13-acetate) was used for the malignant transformation of NE6. Briefly, NE6 was cultured in medium with 10 ng/ml of TPA (12-O-Tetradecanoylphorbol-13-acetate) for 2 weeks, medium was changed every 3 days. After the first round of TPA treatment, NE6 were cultured in normal medium for 2 weeks. Then, the second round of TPA treatment was conducted for another 2 weeks. Totally, NE6 cells were treated by 10 ng/ml TPA for 4 weeks. After TPA treatment, 5% FBS were supplemented in culture medium.

### Histological classifications

Tissues embedded in paraffin were sectioned at 4 μm. H.E. staining was performed for histopathological observation. Normal epithelium was defined as esophageal epithelium with 1–3 basal cell layers. During hyperplasia, over three basal cells layers should be observed or with papillae height over 2/3 of epithelium [14]. Cancer and precancerous lesions diagnosis were given based on the 2010 WHO tumors categorization of the digestive system [20]. For precancerous lesions: low-grade intra-epithelial neoplasia (LGIEN) was defined as when dysplastic epithelial cells were confined to the lower half of the whole epithelium; high-grade intra-epithelial neoplasia (HGIEN) was defined as when dysplastic epithelial cells occupied over half of the whole epithelium. ESCC patients were arranged into Stage I, II, III and IV, according to the 7th Edition of the Union for International Cancer Control-American Joint Committee on Cancer TNM staging system [21].

### Inflammation classification

The degree of chronic inflammation was classified into four degree according to the density and the location of chronic inflammatory cells (lymphocyte and macrophage). *None*, less than 10 inflammatory cells in every high power area restricted to the lamina propria; *mild*, less than 100 inflammatory cells per high power area restricted to the lamina propria, without infiltration in the epithelium; *moderate*, less than 300 inflammatory cells per high power area in lamina propria, less than 20 presented in esophageal epithelium; *severe*, more than 300 inflammatory cells per high power area and more than 20 infiltrated inflammatory cells within epithelium.

### Immunohistochemistry

Antibodies: CD98hc mouse monoclonal antibody was purchased from Santa Cruz#sc-376815 (Dallas, USA), CK14 mouse monoclonal antibody was purchased from Abcam#ab7800 (Cambridge, UK), CK6 rabbit monoclonal antibody was purchased from HUABIO#ET1611-70 (Boston, USA), Ki67 rabbit monoclonal antibody was purchased from MAIXIN Biotechnology #Kit-0005 (Fuzhou, China).

4 μm slides were deparaffinized in xylene, rehydrated through graded ethanol to water. Antigen retrieval was conducted in pressure cooker at 125 °C for 3 min. Endogenous peroxidase was blocked by 3% H_2_O_2_ for 10 min RT. Primary antibodies were applied and incubated at 4 °C in a humidified chamber overnight, followed by incubated with secondary antibody for 30 min at 37 °C. Peroxidase substrate DAB was used for developing. Slides were observed by Olympus BX43 equipped with DP21 digital camera, photographed by CellSens Entry 1.8.

Expression of CD98hc in esophageal epithelium was scored on the percentage taken up by positive stained epithelial cells: score = 0, only the basal layer was of positive; 1, over one layer of the positive cells—25% of the entire epithelium; 2, 26–50%; 3, 51–75%; 4, 76–100%.

The expression of Ki67 in esophageal epithelium was scored based on the ratio of the positive epithelial cells: 0, only one or two layers were positive; 1, more than two layers of positive cells—1/3 of the entire epithelium; 2: 1/3–2/3; 3, > 2/3; 4, the entire epithelium was positive.

### Cell proliferation assay

7500 cells/well in 0.1 ml medium were seeded in 96-well plate, after 48 h, exhausted medium was replaced by 0.1 ml fresh medium, 0.01 ml CCK8 reagents (MCE) were spiked into the medium. The OD value were acquired by iMark plate reader (Bio-Rad). The cell survival rate was calculated according to manufacture guidance.

### Clonal formation assay

100 cells were seeded in 6-well plate, medium was changed every 3 days. After 10 days, cells were fixed by methanol, stained by Giemsa. Photos were taken by digital camera, cell area were measure by ImageJ software 1.52a.

### RT-qPCR analysis

Cellular RNA was obtained by RNeasy Mini kit (Qiagen), gDNA were removed and RNA were reverse transcript to cDNA by Takara#RR047A, qPCR was performed by Takara#RR820A. The primers used were as follows: GAPDH-F 5′-ACAACTTTGGTATCGTGGAAGG, GAPDH-R 5′-GCCATCACGCCACAGTTTC; SLC3A2-F 5′-TGAATGAGTTAGAGCCCGAGA, SLC3A2-R 5′-GTCTTCCGCCACCTTGATCTT.

### Statistical analyses

Statistical analyses were conducted by SPSS 22.0 software (SPSS, Chicago, IL) and GraphPad Prism7. Spearman's rank correlation coefficient analysis was employed for assessing the relevance among chronic inflammation level, esophageal histological severity, expression of CD98hc, expression of Ki67, and histological severity. Chi-square Test and Fisher’s Exact Test were applied on the correlation between CD98hc expression and clinicopathological characteristics. For CCK8 and RT-qPCR results, Shapiro–Wilk normality test and paired T test was utilized in the analysis of the significance of differences. *p* value < 0.05 was considered as statistically significant.

## Results

### CD98hc expression in normal and pathological esophageal epithelium

**Fig. 1:**
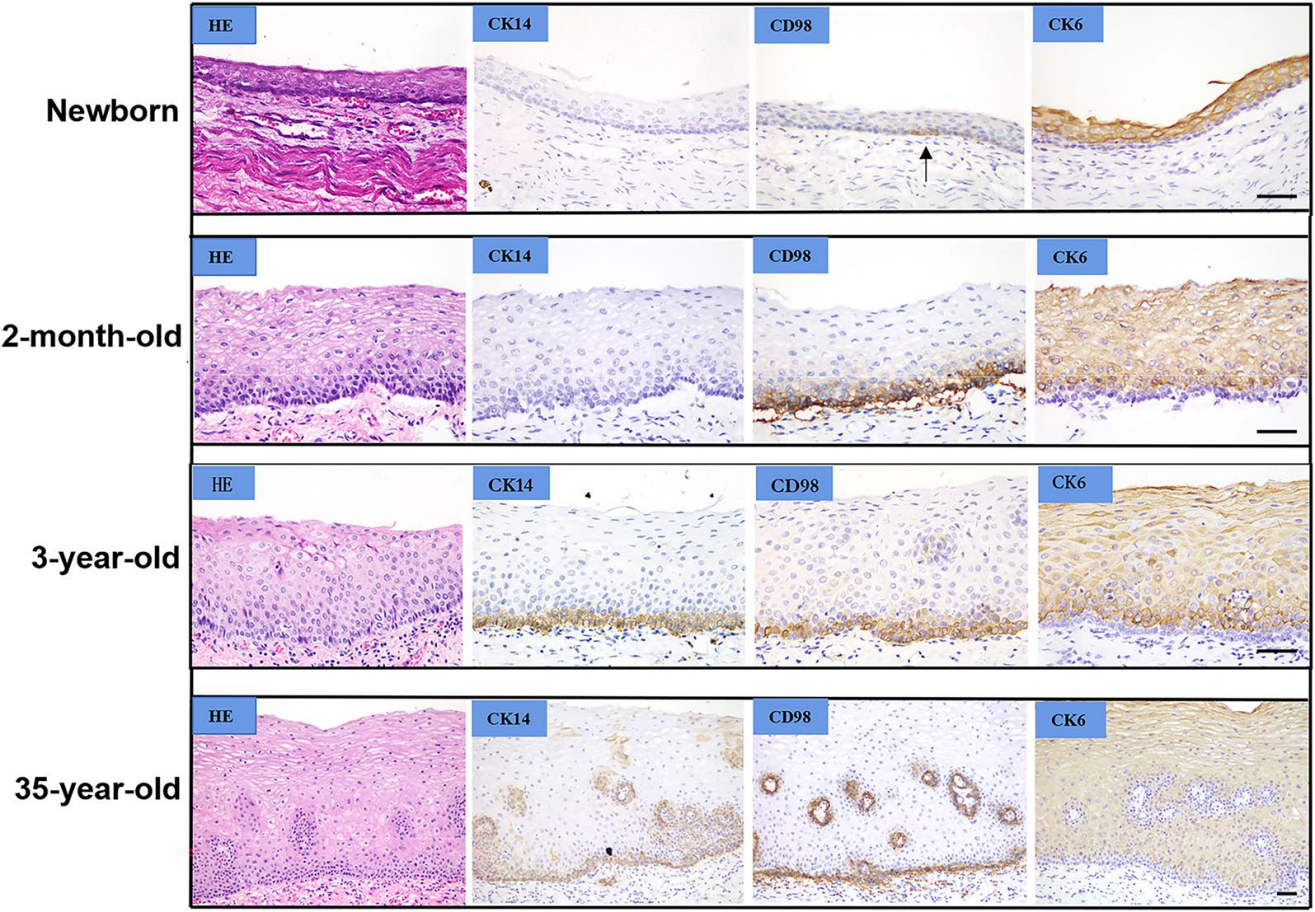
Expression of CK14, CK6 and CD98hc in normal esophageal epithelium from autopsy samples. Scale bar represents 50 μm

**Fig. 2:**
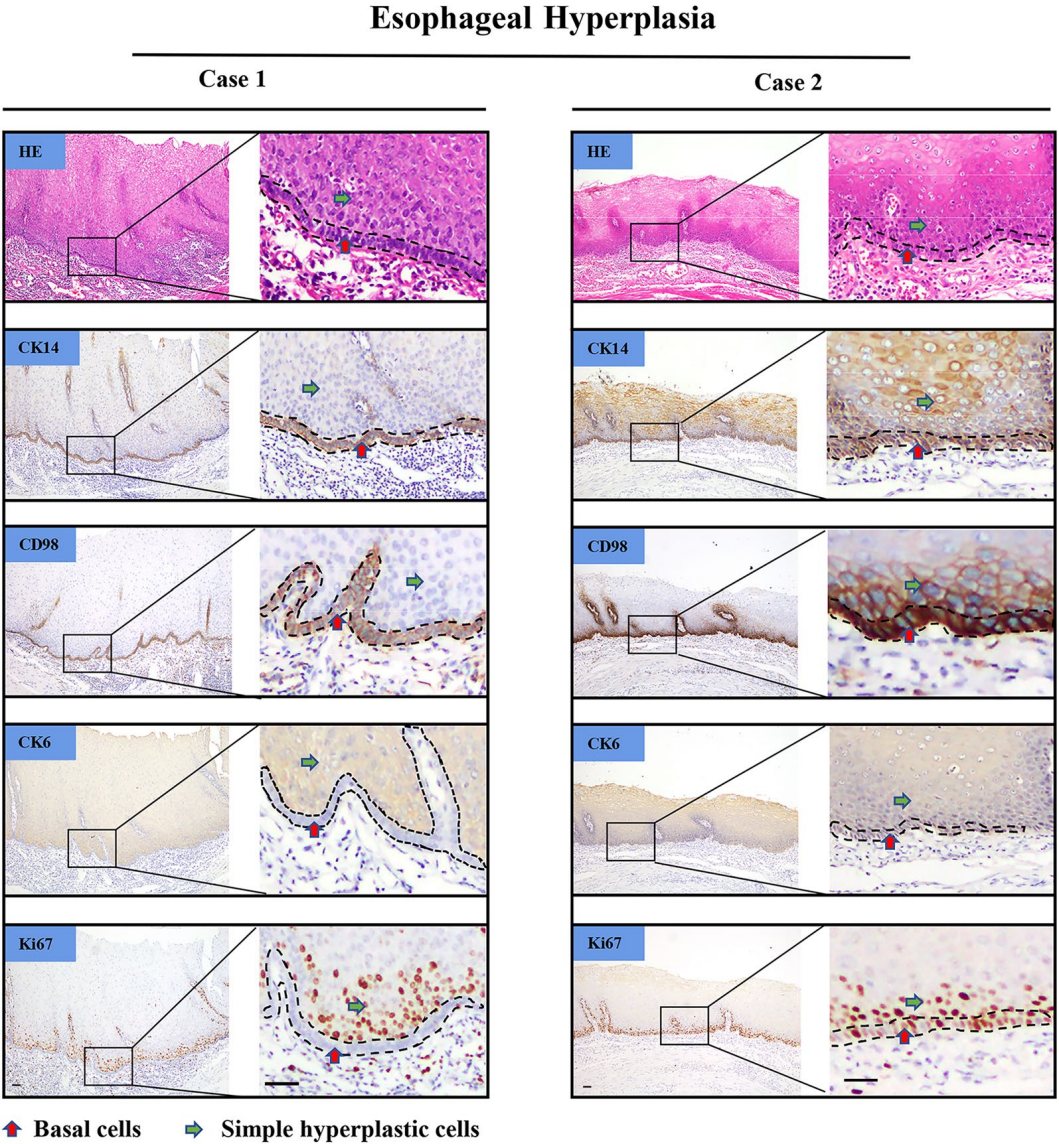
Expression of CK14, CD98hc, CK6 and Ki67 in esophageal hyperplasia. Representative images indicating the existence of two different expression patterns in hyperplastic tissues: basal cells (red arrow head) with CK14 + CD98hc + CK6 − Ki67 − accompanying hyperplastic cells (green arrow head) with CK14 − CD98hc-CK6 + Ki67 + (Case 1); basal cells with CK14 + CD98hc + CK6 + Ki67 + accompanying hyperplastic cells with CK14 + CD98hc + CK6 + Ki67 + (Case 2). Scale bar represents 50 μm

The expression of CD98hc was weak in some cells in the basal layer of fetuses and newborns, and was commonly expressed in basal cells membrane after 2-month-old (Fig. 1, Additional file 2). Photos of IHC staining in esophagus at different ages were not entirely shown. Interestingly, CD98hc expression patterns in ESCC premalignant lesions was not constant. In hyperplastic epithelium, CD98hc expression in basal cells were similar to those in normal epithelium, CD98hc expression could be observed solely in basal cell layer or with the addition of epibasal layer cells accompanied with stronger and more extensive positive signal in cell membrane (Fig. 2).

**Fig. 3:**
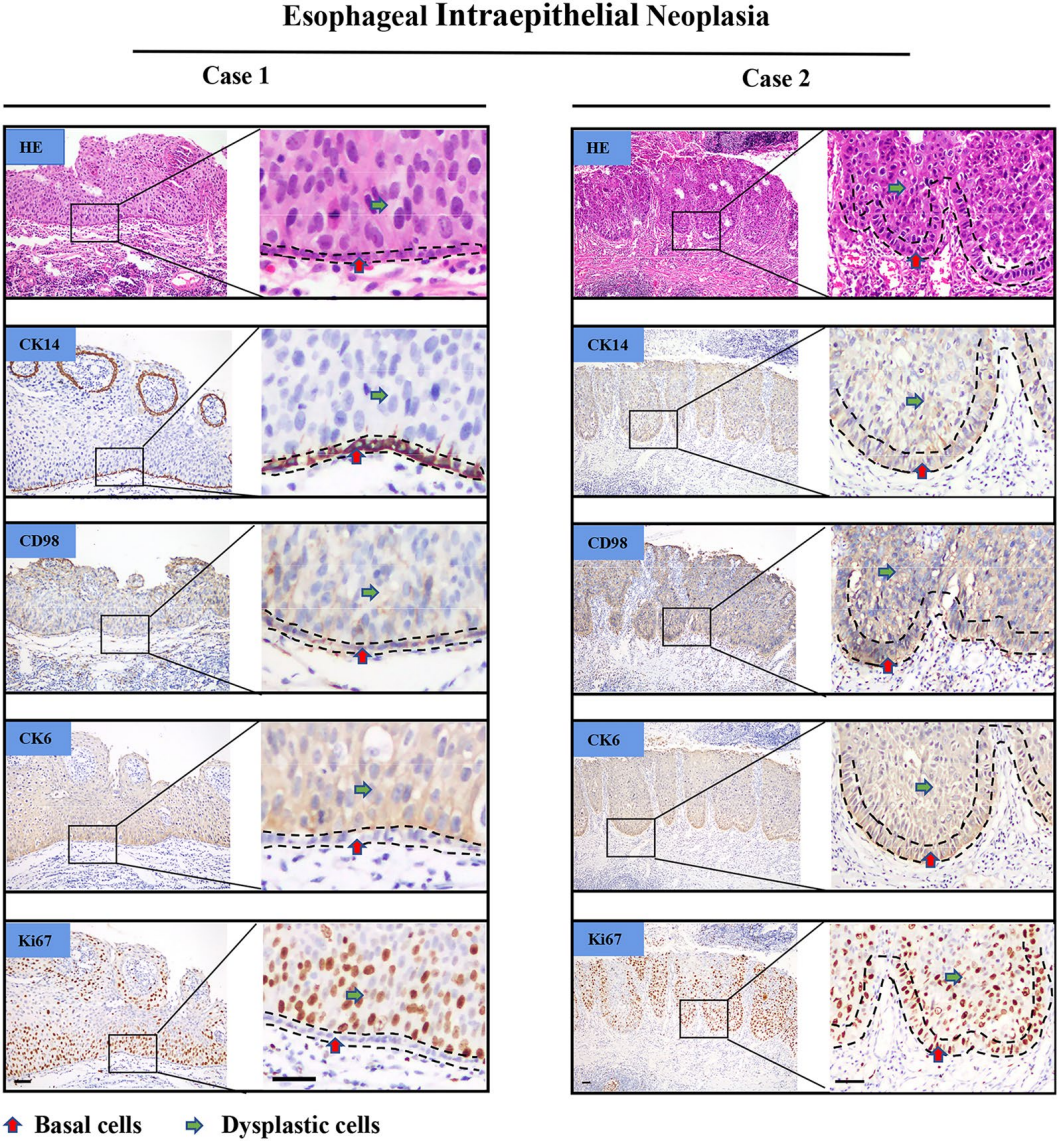
Expression of CK14, CD98hc, CK6 and Ki67 in esophageal intra-epithelial neoplasia. Representative images showing the existence of two different expression patterns in intra-epithelial neoplastic tissues: basal cells (red arrow head) with CK14 + CD98hc + CK6 − Ki67 − accompanying dysplastic cells (green arrow head) with CK14-CD98hc + CK6 + Ki67 + (Case 1); basal cells with CK14 + CD98hc + CK6 + Ki67 + accompanying dysplastic cells with CK14 + CD98hc + CK6 + Ki67 + (Case 2). Scale bar represents 50 μm

In dysplastic epithelium, CD98hc expression was sporadically and weakly observed in basal cell layer, or presented in every dysplastic cells originated from basal cell layer (Fig. 3, case 2). Some of the dysplastic cells that derived from epibasal cell layer mildly expressed CD98hc on the cytomembrane, while normal and hyperplastic cells with the same origination were CD98^**−**^ (Fig. 3, case 1). The above mentioned inconstant CD98hc expression in pathological esophageal epithelium prompted us to investigate the characteristics of those cell referring to their cell of origin and proliferation capacity.

### Proliferated cells were either from basal cells or epibasal cells

By engaging IHC staining against CK14, CD98hc, CK6 and Ki67. We found that in esophageal hyperplastic epithelium, basal cells with CK14^+^CD98hc^+^CK6^−^Ki67^−^ accompanying hyperplastic cells with CK14^−^CD98hc^−^CK6^+^Ki67^+^ occupied 62.5% (15 out of 24) of all samples. Among which, CD98hc were moderately expressed in cell membrane, morphologically, the basal cells were closely and neatly arranged, which could be easily distinguished from upper cells (Fig. 2, Case 1; Additional file 3). Basal cells with CK14^+^CD98hc^+^CK6^+^Ki67^+^ accompanying hyperplastic cells with CK14^+^CD98hc^+^CK6^+^Ki67^+^, however, took up for 25% (6 out of 24). Under this circumstance, CD98hc were strongly expressed in basal cell membrane and gradually decreased to moderate expression in hyperplastic cell above. Basal layer cells lost its neatly arranged structure, was disorderly assembled instead, the morphology and arrangement of basal layer cells were the same as upper cells with no obvious diversity (Fig. 2, Case 2, Additional file 3).

**Fig. 4:**
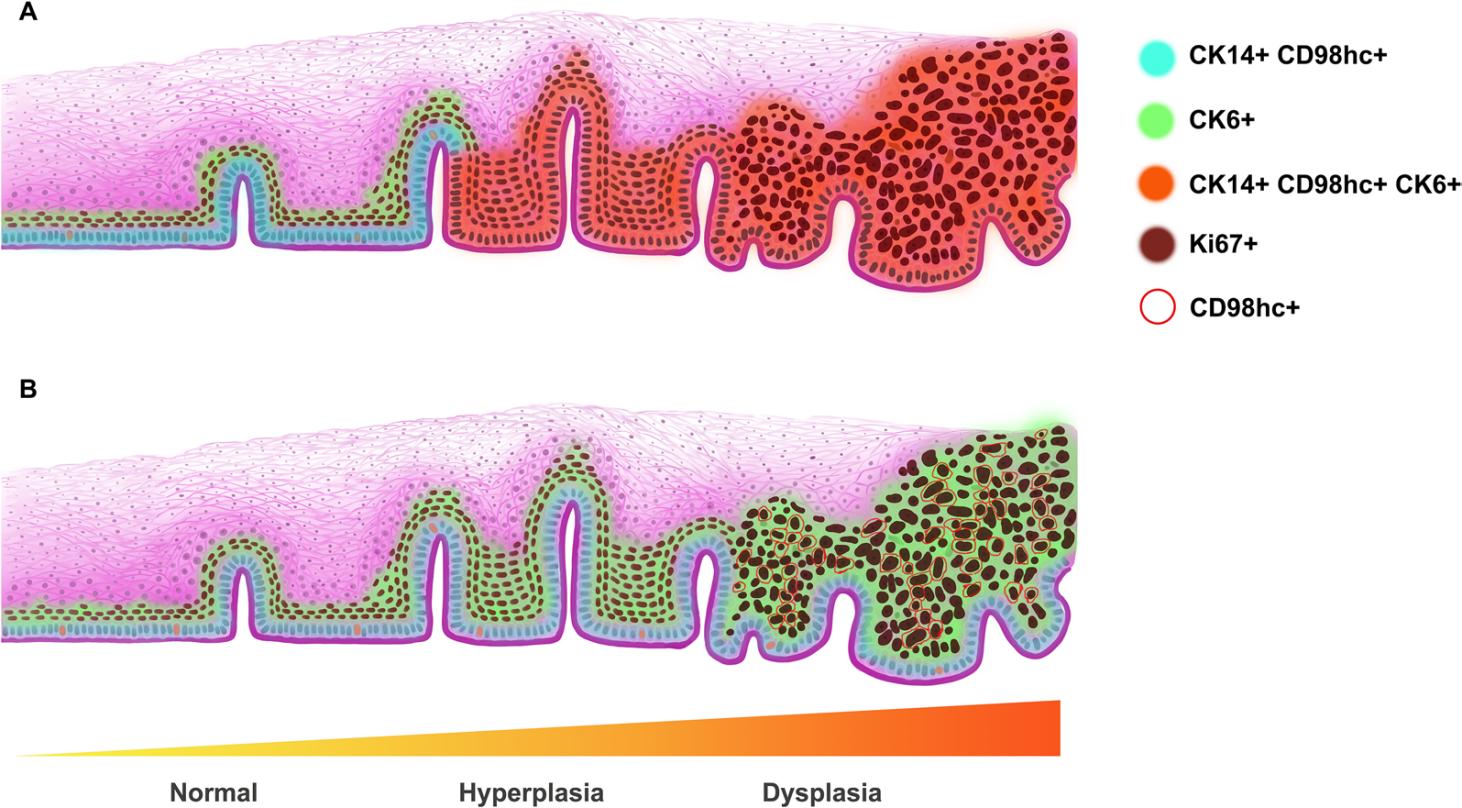
Schema graph demonstrating cell origination in esophageal precancerous lesions. Cells with different expression pattern of CK14, CD98hc and CK6 were covered with different color, Ki67 positive nuclear were in dark brown

In intra-epithelial neoplastic lesions, basal cells with CK14^+^CD98hc^+^CK6^−^Ki67^−^ accompanying dysplastic cells with CK14^−^CD98hc^−^CK6^+^Ki67^+^ accounted for 43.3% (26 out of 60) of all samples. Morphologically, basal cells were arranged in a neat way with roughly consistent shape and were relatively smaller than upper cells (Fig. 3, Case 1, Additional file 3). Basal cells with CK14^+^CD98hc^+^CK6^+^Ki67^+^ accompanying dysplastic cells with CK14^+^CD98hc^+^CK6^+^Ki67^+^ took up for 56.7% (34 out of 60). Basal layer cells were arranged disorderly and resembling the morphology of the upper cells (Fig. 3, Case 2, Additional file 3). Schema graph illustrating the origin of hyperproliferative cells in esophageal precancerous lesions was demonstrated in Fig. 4.

### CD98hc expression was correlated with chronic inflammation, epithelial proliferation capability and premalignant lesion status

**Fig. 5:**
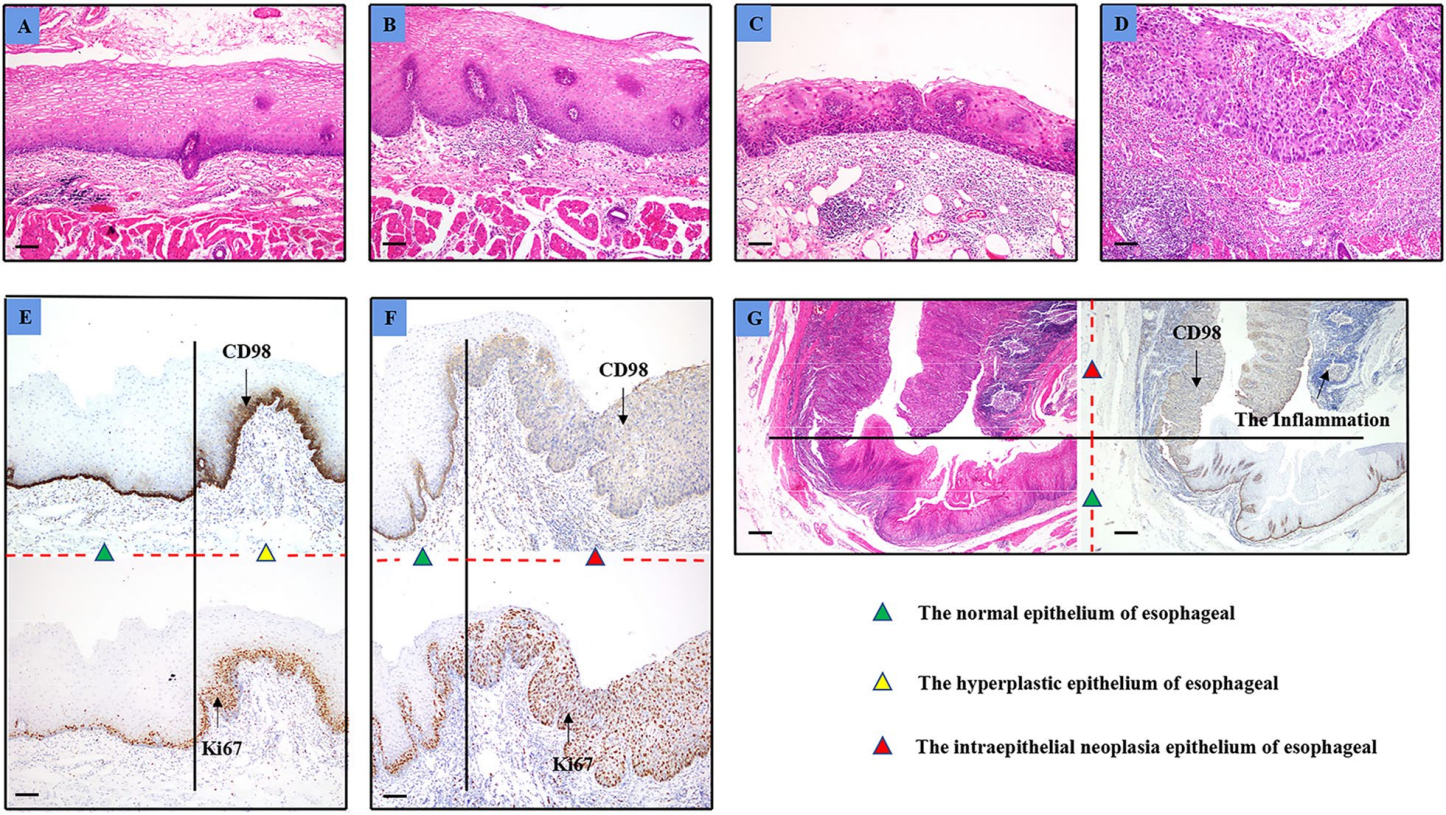
The chronic inflammation degree is relevant to esophageal histological severeness. **A** Representative images expressing normal epithelium without evident inflammation, **B** hyperplastic epithelium with mild inflammation, **C** low-grade intra-epithelial neoplasia epithelium with deep inflammation, **D** high-grade intra-epithelial neoplasia epithelium with severe inflammation. Representative images showing normal esophageal epithelium and esophageal epithelial hyperplasia transition zone (**E**), esophageal intra-epithelial neoplasia and normal esophageal epithelial transition zone (**F**). **G** Representative images showing esophageal intraepithelial neoplasia and normal esophageal epithelial transition zone, with the increase in the expression of CD98hc, the level of inflammation in the lamina propria increases. Scale bar represents 100 μm

In ESCC premalignant lesion samples, CD98hc expression were observed in hyperplastic and dysplastic epithelial cells. Chronic inflammation was commonly observed in esophageal mucosa under pathological conditions, identified by the presence of infiltrating lymphocytes in the epithelium and lamina propria (Fig. 5). According to Spearman's rank correlation coefficient analysis, chronic inflammation status was positively correlated with different pathological changes (r_s_ = 0.804, *p* < 0.001), expression of CD98hc (r_s_ = 0.686, *p* < 0.001), and the presentation of Ki67 (r_s_ = 0.822, *p* < 0.001). Different pathological changes were positively correlated with the expression of CD98hc (r_s_ = 0.840, *p* < 0.001), presentation of Ki67 (r_s_ = 0.941, *p* < 0.001), and the presentation of CD98hc took on positive correlation with the expression of Ki67 (r_s_ = 0.873, *p* < 0.001) (Additional file 1).

### Low dose H_2_O_2_ and TPA stimulated epithelial cell proliferation and upregulated CD98hc expression

**Fig. 6:**
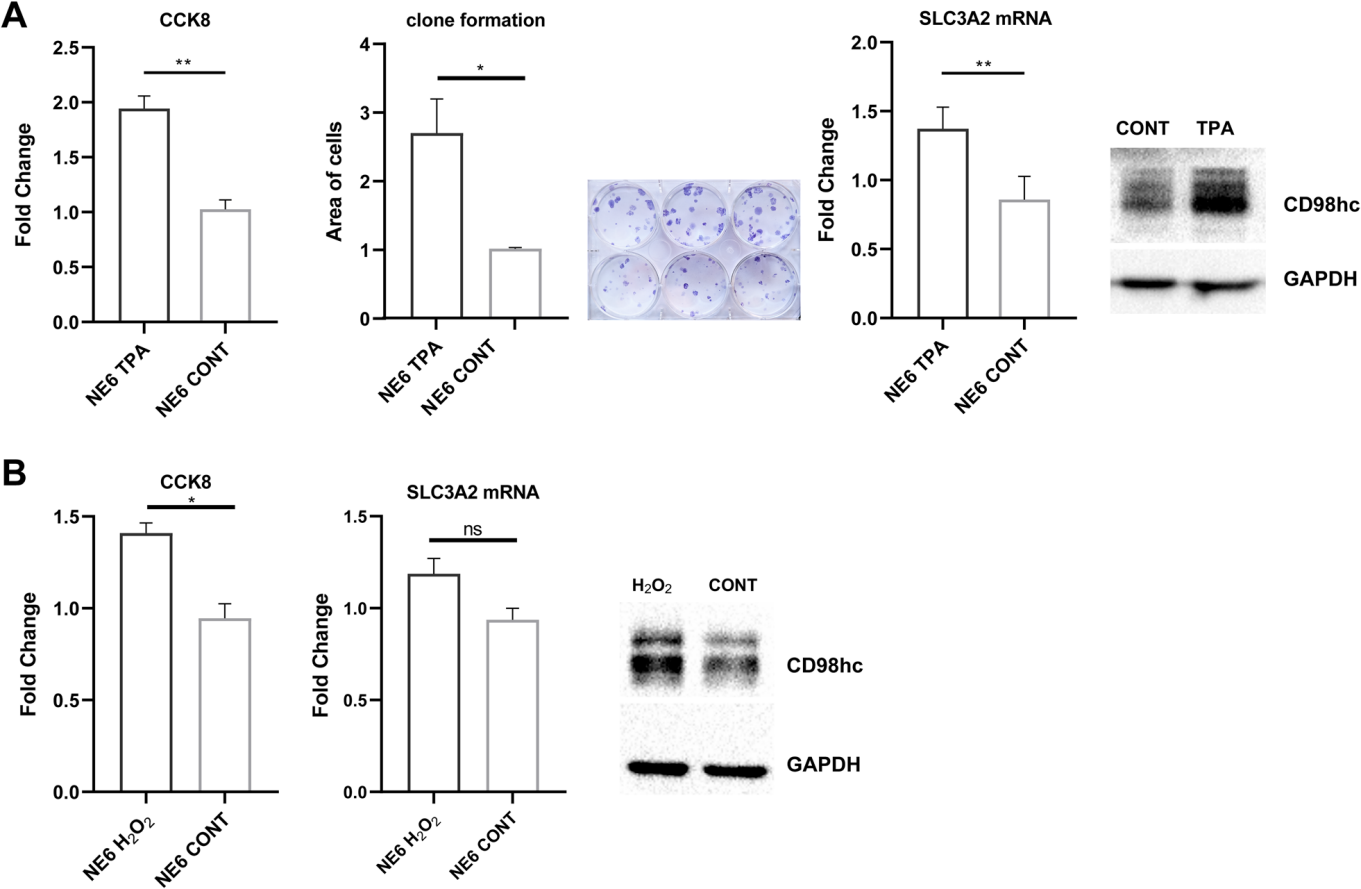
**A** Comparison of cell proliferation and SLC3A2 expression in TPA treated NE6 with control by CCK8 (p = 0.0013), clone formation (p = 0.0286), RT-qPCR (p = 0.0179) and WB. **B** Comparison of cell proliferation and SLC3A2 expression in H2O2 treated NE6 with control by CCK8 (p = 0.015), RT-qPCR (p = ns) and WB

Through the malignant transformation oriented 4 weeks treatment of TPA, the karyotype of NE6 remained diploid which was accessed by metaphase spread (photo not shown). In this case, we considered NE6 was not fully malignanted transformed, but NE6 strain NE6^Hi^ with increased proliferation property was acquired.The proliferation rate of NE6^Hi^ was significantly higher than non-treated cells, revealed by CCK8 and clonal formation assay (Fig. 6A). Meanwhile, CD98hc expression was upregulated as determined by RT-qPCR and WB assay (Fig. 6A). In order to explore the relationship among chronic inflammation, epithelial cell proliferation and CD98hc expression, H_2_O_2_ was utilized to stimulated NE6 cells to mimic reactive oxygen species (ROS) milieu under chronic inflammation condition. After 1 μM H_2_O_2_ treatment for 48 h, the proliferation rate was significantly increased, meanwhile, mRNA and protein level of CD98hc were also upregulated (Fig. 6B).

## Discussion

In normal esophageal epithelium, we found that the expression of CK14 and CD98hc was solely in basal cell layer, while before the age of 2 month, CD98hc only expressed separately in basal cells, and given that papillae were not observed before the age of 3 (photo not shown), we proposed CD98hc being a specific marker for mature esophageal basal cells.

By engaging IHC staining, we identified the proliferated cells in hyperplastic and dysplastic esophageal epithelium were either derived from basal cells or epibasal cells. We have also observed that proliferated cell originated from basal cell exhibited higher expression intensity of CD98hc than that of cell derived from epibasal cell. The difference in CD98hc expression intensity might reflect their unequal demands of specific energy and biosynthesis [22]. Basal cell layer is the polarized bottom cell layer of esophageal epithelium, which firmly seated above the thin BM. Basal cells are capable of mitosis to produce daughter cell for epithelium renewal. Their normal function depending on polarity, to some extent, is control by integrin β1 [23, 24]. Integrin β1 is a major component of focal adhesion, a transmembrane structure that adheres basal cells with BM [8]. Gilles Lemaître et al. had demonstrated that CD98hc regulate keratinocyte adhesion by interacting with integrin β1 [8]. Since CD98hc not only served as amino acid transporter, but also regulate some biological processes, the potential role of CD98hc other than its transporter character in the growth and malignant differentiation aspect of dysplastic cells is worthy of further studying. The expression of CD98hc exhibited ectopic feature as well. CD98hc was expressed on cytomembrane in hyperplastic cells and normal epithelium, apart from cytomembrane, CD98hc was also expressed in cytoplasm in dysplastic cells. As our group has reported before, dysplastic cells were genetically similar with cancer cells but different from hyperplastic cells [11]. The ectopic expression of CD98hc in dysplastic cell also indicated the distinct biological property between dysplastic and hyperplastic cells. From another point of view, the ectopic expression of CD98hc in dysplastic cells might imply CD98hc has other roles in maintaining the growth of dysplastic cells. Interestingly, in some dysplastic cell derived from epibasal cells that supposed to be CD98hc^**−**^ were mild positive, this de novo expression of CD98hc hinted the importance of CD98hc in the initiation of ESCC.

Our in vitro assays utilizing immortalized esophageal epithelial cell line demonstrated that low dose ROS could increase CD98hc expression and proliferation, carcinogen treated cell possessed upregulated CD98hc expression and proliferation rate. In accordance with our IHC results, CD98hc expression was correlated with chronic inflammation, epithelial proliferation capability and premalignant lesion status. CD98hc has been shown to regulate cell proliferation by MAPK pathways in renal epithelial cell [25]. CD98hc could endowed tumorigenic characteristics to cancer cells through its integrin binding domain in clear cell renal cancer and skin cancer [26, 27]. Moreover, CD98hc was also implicated as regulator of CD4 + T cells and IFN-γproduction, both of which play a role in anti-tumor immunity [28].

Thus, we proposed a hypothesis that CD98hc expression in normal esophageal epithelium basal cells could maintain their polarity and adhesion with BM, when basal cells exhibit abnormal CD98hc expression referring to the intensity and cellular location, basal cells lost adhesion with BM, outside-in signal transduction and polarity, which result in uncontrolled proliferation and anchorage independent growth favoring carcinogenesis.

To sum up, the current study demonstrated that hyperplastic/dysplastic cells in esophageal epithelium were originated from basal or epibasal cells, revealed by the expression of CD98hc. And we proposed that ectopic expression of CD98hc in hyperplastic/dysplastic cells was induced by chronic inflammation stimulation, which crippled the linkage between basal cell and BM, sabotaged the integrity of the barrier in between lamina propria and epithelium, subsequentially initiate carcinogenesis. We emphasized that further studying about of the role of CD98hc in ESCC initiation and progression could shed new light on the early diagnosis, treatment and prognostic evaluation of ESCC.

## Supplementary Information

**Additional file 1. **Correlation analysis revealed the existence of a significant positive correlation between any two of the histological severity of esophagus, chronic inflammation, expression of CD98hc and expression of Ki67.

**Additional file 2. **Expression of CK14, CK6 and CD98hc in esophageal epithelium at different ages.

**Additional file 3. **Expression of CK14, CD98hc, CK6 and Ki67 in esophageal hyperplasia and esophageal intraepithelial neoplasia.

## Data Availability

Additional data were in additional files.

## Abbreviations

ESCC: Esophageal squamous cell carcinoma
LGIEN: Low-grade intra-epithelial neoplasia
HGIEN: High-grade intra-epithelial neoplasia
BM: Basement membrane
IHC: Immunohistochemistry
TPA: 12-O-Tetradecanoylphorbol-13-acetate
STR: Short tandem repeats
DAB: Diaminobenzidine
