# Supplementary figures and images for: CD98hc has a pivotal role in maintaining the immuno-barrier integrity of basal layer cells in esophageal epithelium
Source: Cancer Cell Int. 2022 Feb 22;22:98. doi: 10.1186/s12935-021-02399-5 (PMC8864845; doi:10.1186/s12935-021-02399-5)

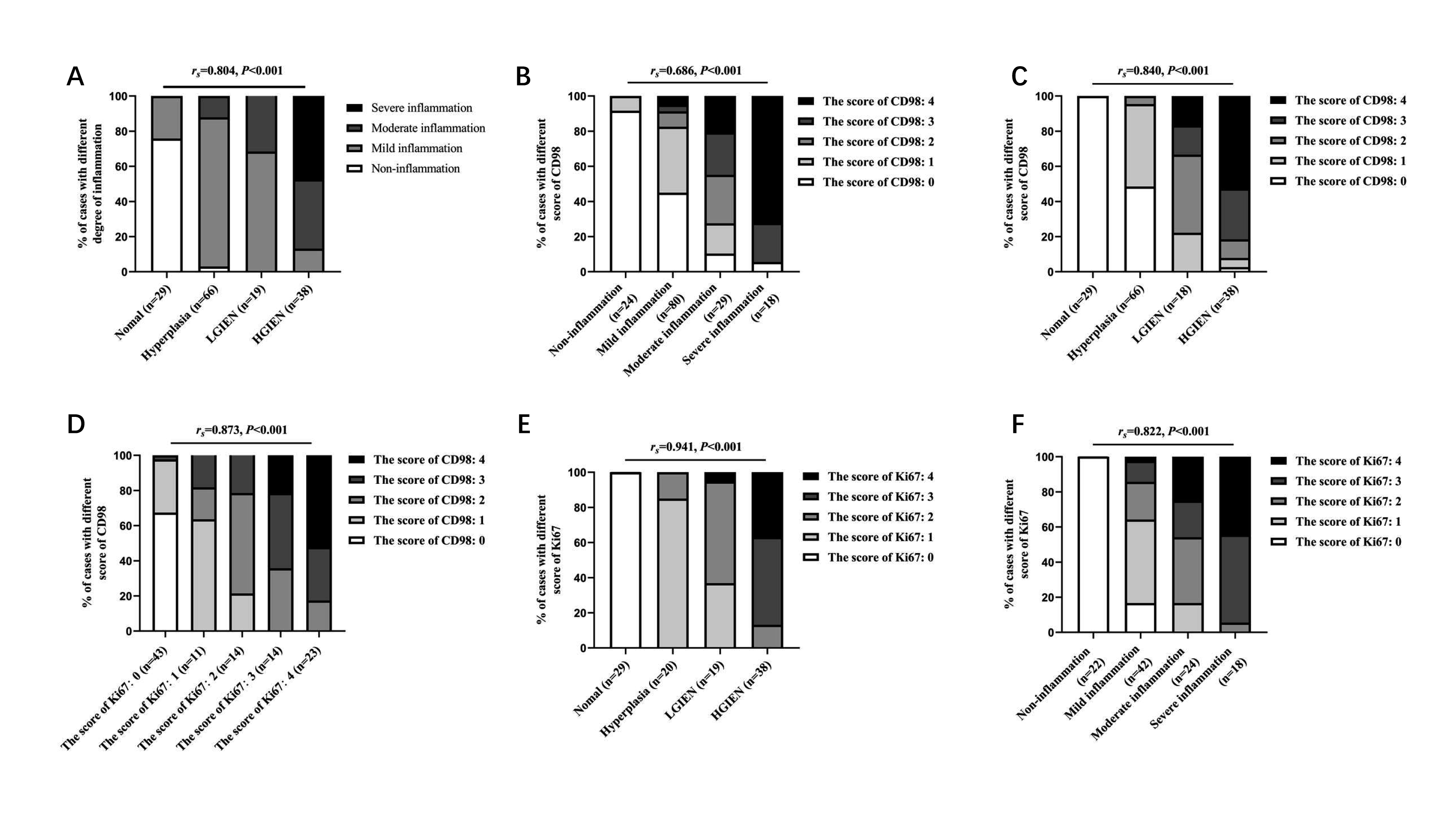

Supplement: Supplementary file 1 — Additional file 1. Correlation analysis revealed the existence of a significant positive correlation between any two of the histological severity of esophagus, chronic inflammation, expression of CD98hc and expression of Ki67. [file 12935_2021_2399_MOESM1_ESM.tif]
