# Supplementary material for: CD98hc has a pivotal role in maintaining the immuno-barrier integrity of basal layer cells in esophageal epithelium
Source: Cancer Cell Int. 2022 Feb 22;22:98. doi: 10.1186/s12935-021-02399-5 (PMC8864845; doi:10.1186/s12935-021-02399-5)
Supplement: Supplementary file 2 — Additional file 2. Expression of CK14, CK6 and CD98hc in esophageal epithelium at different ages. [file 12935_2021_2399_MOESM2_ESM.docx]

| Age | Type of cell | CK14 | CD98hc | CK6 |
| --- | --- | --- | --- | --- |
| Fetus (n=2) | Basal cells | - | +/- | - |
|  | Suprabasal cells | - | - | + |
| Newborn (n=3) | Basal cells | - | +/- | - |
|  | Suprabasal cells | - | - | + |
| 2m (n=1) | Basal cells | - | + | - |
|  | Suprabasal cells | - | - | + |
| 1.5y (n=1)  3y (n=3)  7y (n=1)  9y (n=1)  15y (n=1)  18y (n=1)  28y (n=1)  35y (n=1)  47y (n=1)  56y (n=1)  63y (n=1)  73y (n=1)  82y (n=1) | Basal cells | + | + | - |
|  | Suprabasal cells | - | - | + |

**Additional file 2**
