# Supplementary material for: CD98hc has a pivotal role in maintaining the immuno-barrier integrity of basal layer cells in esophageal epithelium
Source: Cancer Cell Int. 2022 Feb 22;22:98. doi: 10.1186/s12935-021-02399-5 (PMC8864845; doi:10.1186/s12935-021-02399-5)
Supplement: Supplementary file 3 — Additional file 3. Expression of CK14, CD98hc, CK6 and Ki67 in esophageal hyperplasia and esophageal intraepithelial neoplasia. [file 12935_2021_2399_MOESM3_ESM.docx]

|  | | | CK14 | CD98hc | CK6 | Ki67 |
| --- | --- | --- | --- | --- | --- | --- |
| Esophageal  hyperplasia (n=24) | 15/24 | Basal cells | + | + | - | - |
|  |  | Simple hyperplastic cells | - | - | + | + |
|  | 6/24 | Basal cells | + | + | + | + |
|  |  | Simple hyperplastic cells | + | - | + | + |
| Esophageal intraepithelial  neoplasia (n=60) | 26/60 | Basal cells | + | + | - | - |
|  |  | Dysplastic cells | - | + | + | + |
|  | 34/60 | Basal cells | + | + | + | + |
|  |  | Dysplastic cells | + | + | + | + |

**Additional file 3**
